# Docetaxel-Loaded Nanoparticles Assembled from β-Cyclodextrin/Calixarene Giant Surfactants: Physicochemical Properties and Cytotoxic Effect in Prostate Cancer and Glioblastoma Cells

**DOI:** 10.3389/fphar.2017.00249

**Published:** 2017-05-08

**Authors:** Laura Gallego-Yerga, Inmaculada Posadas, Cristina de la Torre, Jesús Ruiz-Almansa, Francesco Sansone, Carmen Ortiz Mellet, Alessandro Casnati, José M. García Fernández, Valentín Ceña

**Affiliations:** ^1^Departamento de Química Orgánica, Facultad de Química, Universidad de SevillaSevilla, Spain; ^2^CIBERNED, Instituto de Salud Carlos IIIMadrid, Spain; ^3^Unidad Asociada Neurodeath, Facultad de Medicina, Universidad de Castilla-La ManchaAlbacete, Spain; ^4^Dipartimento di Chimica, Università degli Studi di ParmaParma, Italy; ^5^Instituto de Investigaciones Químicas, CSIC – Universidad de SevillaSevilla, Spain

**Keywords:** prostate cancer, cyclodextrins, calixarenes, docetaxel, glioblastoma

## Abstract

Giant amphiphiles encompassing a hydrophilic β-cyclodextrin (βCD) component and a hydrophobic calix[4]arene (CA_4_) module undergo self-assembly in aqueous media to afford core-shell nanospheres or nanocapsules, depending on the nanoprecipitation protocol, with high docetaxel (DTX) loading capacity. The blank and loaded nanoparticles have been fully characterized by dynamic light scattering (DLS), ζ-potential measurements and cryo-transmission electron microscopy (cryo-TEM). The data are compatible with the distribution of the drug between the nanoparticle core and the shell, where it is probably anchored by inclusion of the DTX aromatic moieties in βCD cavities. Indeed, the release kinetics profiles evidenced an initial fast release of the drug, which likely accounts for the fraction hosted on the surface, followed by a slow and sustained release rate, corresponding to diffusion of DTX in the core, which can be finely tuned by modification of the giant amphiphile chemical structure. The ability of the docetaxel-loaded nanoparticles to induce cellular death in different prostate (human LnCap and PC3) and glioblastoma (human U87 and rat C6) cells was also explored. Giant amphiphile-based DTX formulations surpassing or matching the antitumoral activity of the free DTX formulation were identified in all cases with no need to employ any organic co-solvent, thus overcoming the DTX water solubility problems. Moreover, the presence of the βCD shell at the surface of the assemblies is intended to impart stealth properties against serum proteins while permitting nanoparticle surface decoration by supramolecular approaches, paving the way for a new generation of molecularly well-defined antitumoral drug delivery systems with improved specificity and efficiency. Altogether, the results provide a proof of concept of the suitability of the approach based on βCD-CA_4_ giant amphiphiles to access DTX carriers with tunable properties.

## Introduction

Prostate cancer (PCa) is the most commonly diagnosed cancer in men and it is one of the leading causes of death worldwide (Zhou et al., 2016). In the early-stage, PCa is androgen-dependent for growth and survival, and androgen ablation therapy usually causes its regression. However, some of the PCas evolve to aggressive and drug-resistant tumors. Docetaxel (DTX) is a second-generation cytotoxic agent derived from taxol which has been proven to have significant antitumor activity against various human cancers, including PCa (Sweeney et al., 2015; James et al., 2016), metastasized glioblastoma (Astner et al., 2006), HER-2 positive metastatic breast cancer (Swain et al., 2015) and lung cancer (Shaw et al., 2013). Docetaxel binds preferentially to the tubulin β-subunit, stabilizing the microtubules and inhibiting depolymerization, which leads to cell cycle arrest, mainly in the G2/M phase, and, ultimately, to cell death by apoptosis (Montero et al., 2005). Regrettably, resistance to docetaxel appears frequently during therapy, possibly involving several mechanisms such as over expression of drug efflux pumps, acquired mutations of the drug binding site in tubulin or activation of growth factor survival pathways (Dumontet and Sikic, 1999; Gottesman et al., 2002), which becomes a major limitation for the therapeutic use of the drug (Giannakakou et al., 2000; He et al., 2001).

Due to its poor aqueous solubility (10–20 mM in plain water), DTX is currently formulated in polysorbate 80 (Taxotere^®^) in anhydrous form, since DTX degrades over time in protic solvents (Rao et al., 2006). This formulation is known to cause severe allergic reactions and peripheral neuropathy (ten Tije et al., 2003) the incidence of hypersensitivity ranging from 5 to 40%. Moreover, after dilution with the 13% hydroethanolic vehicle provided, the adverse effects may be intensified and the formulation becomes physically unstable and must be administered to the patient within 8 h. Investigational approaches have focused in macromolecular multiconjugates or nanoparticles as solubilizing and delivery agents since such constructs generally protect DTX from degradations, benefit from passive targeting to tumoral tissues by enhanced permeability and retention (EPR) effect, and can be modified with specific groups for tumor-specific targeting (Zhang and Zhang, 2013). Examples of nanometric platforms used in the design of DTX formulations on record include low molecular weight chitosan (Lee et al., 2009), dendrimers (Gajbhiye and Jain, 2011), lipid-based formulations (Ren et al., 2016), C60 fullerene (Raza et al., 2015) and gold nanoparticles (Francois et al., 2011). Whereas significant improvements in drug biodistribution and tumoricidal efficiency have been reported, the intrinsic polydispersity of such systems represents a limitation for structure-activity relationship (SAR) and optimization studies that may seriously hamper translation into hospital settings.

An ideal DTX formulation should rely on molecularly well-defined vehicles, susceptible of physicochemical tailoring in order to impart biocompatibility, efficiency to the target cells, and high drug loading with appropriate drug release characteristics, thereby preventing drug inefficiency and side effects. Precision macromolecular synthesis represents a unique approach for those purposes, since it allows engineering structures across multiple length scales with accurate control of their self-assembling and macroscopic properties, and offers considerable potential for the encapsulation, delivery and controlled release of pharmaceuticals. This goal can be realized by linking shape- and volume-persistent nano-objects with a well-defined molecular structure and specific symmetry, generically termed molecular nanoparticles (MNPs). The control of hierarchical structures from the resulting “giant molecules” can then be facilitated by tuning the collective physical interactions between the relatively independent nanosized subunits. Among giant molecules, giant surfactants have shown self-assembling properties that are extremely sensitive to topological variations, providing unprecedented opportunities for the design and programming of advanced materials possessing a specific functionality (Yu et al., 2013).

In a preliminary publication, we previewed an original giant surfactant prototype based on β-cyclodextrin (βCD) and calix[4]arene (CA_4_) heterodimers with the capability to self-assemble into core-shell nanosystems with drug encapsulation and controlled release capabilities (Gallego-Yerga et al., 2014). βCD, the most accessible representative of the cyclodextrin (CD) family, is a water-soluble macrocyclic compound made of seven α(1,4)-linked glucose units that feature a truncated-cone shape with an external hydrophilic surface and a hydrophobic cavity that can host different molecules and transport them in biological media (Kurkov and Loftsson, 2013; James et al., 2016). Interestingly, CDs can be chemically modified and decorated with targeting groups, which has been exploited for site-specific drug delivery to different cell types, such as macrophages (Benito et al., 2004). Of particular interest for our goals is the fact that βCD can form inclusion complexes with docetaxel and that the incorporation of βCD in nanomaterials can be used to impart high DTX loading capabilities (Zhao and Astruc, 2012; Wang et al., 2016). Although canonic βCD exhibit some renal toxicity after parenteral administration, related to its greater ability to interact with cellular lipids leading to cell-membrane damage, toxicity is greatly reduced after chemical modification (Stella and He, 2008; Gallego-Yerga et al., 2015a). Indeed, amphiphilic βCD derivatives showed no apparent toxicity in animal models (Mendez-Ardoy et al., 2011; Aranda et al., 2013; Gallego-Yerga et al., 2015a).

Similarly, calixarenes (CAs) are macrocyclic molecules formed by *para*-substituted phenol units linked through methylene bridges that likewise form a cavity that can host cations, amino acids or peptides (Sansone et al., 2010). Although DTX does not match the cavity size of calixarene hosts, nanocarriers constructed from amphiphilic calix[4]arene derivatives have shown excellent taxane drug loading capabilities (Weeden et al., 2012). The combination of βCD and CA_4_ elements in a single macromolecule has therefore the potential of benefiting from the favorable properties of both entities for DTX transport and delivery (Wang et al., 2013).

**FIGURE 1:**
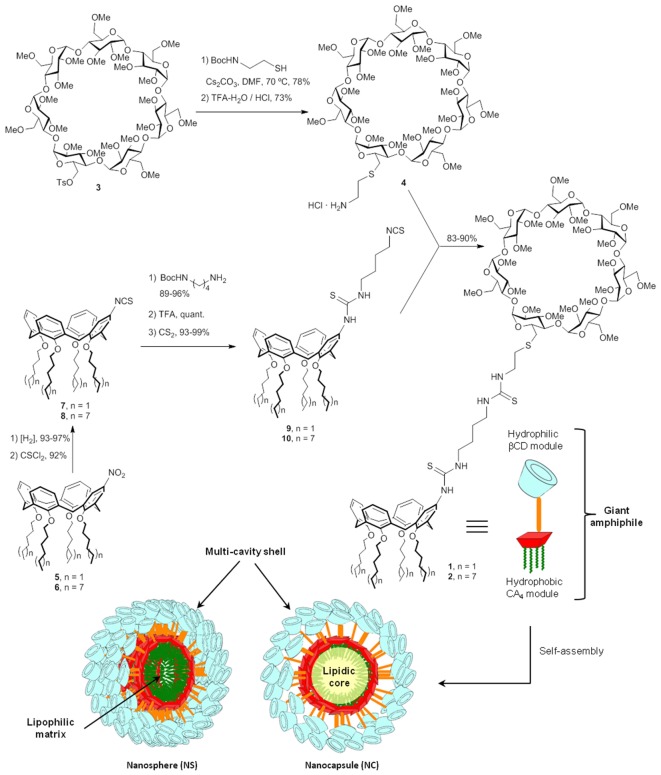
**Synthesis of the CA_4_ - βCD giant surfactants and schematic representation of their self-assembly into nanospheres and nanocapsules**.

Herein we report the preparation of self-assembled nanocapsules (NCs) and nanospheres (NSs) from the giant surfactants **1** and **2**, obtained by “click”-type heterodimerization of regioselectively functionalized hydrophilic βCD and hydrophobic CA_4_ MNPs (**Figure 1**). The resulting multicavity systems have been characterized in terms of their hydrodynamic diameter, ζ-potential and morphology by dynamic light scattering, electrophoretic mobility and cryo-transmission electron microscopy techniques. After DTX-loading, the resulting formulations have shown efficient cytotoxic activity in two human cell lines of PCa (LnCap and PC3) and in two glioblastoma cell lines of human (U87) and murine (C6) origin. Altogether the results demonstrate the suitability of the approach based on βCD-CA_4_ hybrid molecules to generate DTX nanocarriers with varied properties. Moreover, the fact that the best performing formulation in terms of cytotoxicity depends on the target cell line highlights the importance of developing strategies that not only keep full control on the chemical structure of the MNP, but that are also molecular diversity-oriented and compatible with SAR analysis. Previous reports supporting that nanoencapsulation of DTX bears considerable promise for the development of oral formulations represent a further motivation for this research (Attili-Qadri et al., 2013; Cho et al., 2014). Nevertheless, ADME-Tox and pharmacokinetic studies are out of the scope of the present work.

## Materials and Methods

### Synthesis and Characterization

The CA_4_-βCD giant surfactants **1** and **2** were obtained by high-yielding “click”-type thiourea coupling reactions between the isothiocyanate-armed tetraalkylated CA_4_ derivative **4** or **5** and the amine-equipped βCD derivative **3** (**Figure 1**), using a previously optimized semi-convergent synthetic strategy (Gallego-Yerga et al., 2014). The βCD precursor **3** was prepared in 92% yield from the known mono-O6-tosyl-derivative **3** (Kaneda et al., 2002) after reaction with Boc-protected cysteamine and final acid-catalyzed hydrolysis (→**4**). The synthesis of the CA_4_ counterparts started from the singly functionalized derivatives **5** and **6**, accessed by mononitration of the corresponding lower-ring tetra-hexyl and -dodecyl ethers, which proceeded in 75% after treatment with concentrated nitric acid in a mixture of dichloromethane and glacial acetic acid (Keldermann et al., 1992). Sequential reduction to the corresponding amines followed by isothiocyanation afforded the intermediates **7** and **8**. Thiourea coupling with mono-Boc-protected butylethylenediamine, carbamate hydrolysis and final isothiocyanation proceeded with over 90% yield in every step. Thiourea conjugation of βCD **4** with the CA_4_ partners **7** and **8** provided the target heterodimers **1** and **2** in 83 and 90% yield, respectively.

#### Hydrodynamic Diameter and ζ-Potential Measurements

The average sizes of the self-assembled nanoparticles (NSs or NCs) were measured using a *Zetasizer Nano ZS* (*Malvern Instruments*) with the following specifications: sampling time, automatic; number of measurements, 3 per sample; medium viscosity, 1.054 cP; refractive index, 1.33; scattering angle, 173°; λ = 633 nm; temperature, 25°C. Data were analyzed using the multimodal number distribution software included in the instrument. ζ-Potentials were determined using the “mixed-mode measurement” phase analysis light scattering (M3-PALS) following the specifications used for hydrodynamic diameter measurements. Before each series of experiments, the performance of the instruments was checked with either 90-nm monodisperse latex beads (Coulter) for DLS or with DTS 50 standard solution (Malvern) for ζ-potentials.

#### Cryo-Transmission Electron Microscopy (Cryo-TEM)

The transmission electron microscope used to obtain cryo-TEM micrographs of blank and DTX-loaded NSs and NCs was a JEOL JEM 1400 instrument operating at 120 kV. To prepare the samples, 5 μL of the NS or NC suspension was deposed on a QUANTIFOIL^®^ R 1.2/1.3 grid and the excess was eliminated with Whatman N°1 paper. Vitrification was done with a CPC Leica by immersing the grid as fast as possible in liquid ethane. The measurements were carried out at the facilities of the Center for Biological Research (CSIC, Madrid, Spain).

#### Preparation of Unloaded (Blank) Nanospheres

Blank NS suspensions were prepared using the nanoprecipitation technique (Skiba et al., 1996) by taking advantage of the spontaneous self-assembling capabilities of the amphiphilic βCD-calixarene heterodimer derivatives **1** and **2** when dispersed into an aqueous phase. Briefly, the corresponding giant surfactant species was dissolved in anhydrous methanol to a final concentration of 0.4 mM, and this solution was dropwise added into an equal volume of milliQ water containing a non-ionic hydrophilic surfactant (*Polysorbate 80*, 2 mg⋅mL^-1^), under magnetic stirring (300 rpm) at 25°C for 5 min. Nanoparticles were formed spontaneously and the organic solvent was removed under reduced pressure at 35°C. The nanoparticle suspensions, prepared in triplicate, were subjected to centrifugation (1,957 × *g*, 15 min) to remove traces of any aggregated giant amphiphile and stored in closed vials at +4°C.

#### Preparation of Docetaxel-Loaded Nanospheres

Docetaxel-loaded NS formulations were prepared using the procedure described above, but starting from methanol solutions containing both the corresponding amphiphilic derivative **1** or **2** (0.4 mM) and DTX (1.2 mM). The aqueous NS suspensions, prepared in triplicate, were subjected to centrifugation (1,957 × *g* rpm, 15 min) to remove traces of unloaded (insoluble) docetaxel that might potentially precipitate or any aggregated giant amphiphile and stored in closed vials at 4°C.

#### Preparation of Unloaded (Blank) Nanocapsules

The corresponding giant surfactant **1** or **2** was dissolved in anhydrous methanol at 0.4 mM containing a small amount of capric/caprylic triglycerides (*Labrafac Lipophile WL1349*, 2 mg⋅mL^-1^) and a non-ionic hydrophobic surfactant (*Span 80*, 2 mg⋅mL^-1^). This solution was added dropwise into an equal volume of milli-Q water containing a non-ionic hydrophilic surfactant (*Polysorbate 80*, 2 mg⋅mL^-1^), under magnetic stirring (300 rpm) at 25°C for 5 min. Blank NCs were thus formed spontaneously. The organic solvent was removed under reduced pressure at 35°C and the resulting aqueous suspensions, prepared in triplicate, were subjected to centrifugation (1,957 × *g*, 15 min) to remove traces of any aggregated giant amphiphile and stored in closed vials at +4°C.

#### Preparation of Docetaxel-Loaded Nanocapsules

Docetaxel-loaded NC suspensions were prepared using the procedure described above, but starting with methanol solutions containing both the corresponding amphiphilic derivative **1** or **2** (0.4 mM) and docetaxel (1.2 mM). The NC suspensions, prepared in triplicate, were subjected to centrifugation (1,957 × *g*, 15 min) to remove traces of unloaded (insoluble) docetaxel or aggregated giant amphiphile and stored in closed vials at +4°C.

#### Determination of DTX Encapsulation Efficiency and Loading Capacity

The supernatant obtained after centrifugation (1,957 × *g*, 15 min) of the corresponding DXT-loaded NSs or NCs suspensions was freeze-dried, solubilized in methanol and the DTX concentration determined spectrophotometrically (Jasco UV-630 spectrometer) at 230 nm and 25°C by absorbance interpolation in a calibration plot. We run control experiments to ensure that the giant amphiphiles **1** or **2** did not interfere in determination of encapsulated DTX. Indeed, the βCD unit does not absorb in the UV and the calix moiety have two absorption maxima at 280 and 288 nm, far enough from the 230 nm maximum of DTX. The encapsulation efficiency (EE; %) was determined as the quotient (w/w; x100) between the entrapped drug (i.e., total minus unbound drug) and the total drug used in the formulation. The loading capacity LC (%) is obtained as the quotient (w/w; x100) between the entrapped drug and the total amount of encapsulated drug and giant amphiphile used in the formulation.

#### *In Vitro* Docetaxel Release Kinetics

*In vitro* DTX release kinetics studies were performed by dialysis (cutoff of 12 kDa, Sigma) in a phosphate buffer saline medium (PBS, 0.01 M, pH 7.3, 37 °C). DTX-loaded NS or NC suspensions, prepared as described above, were dialyzed against 125 mL of PBS under smooth stirring. Aliquots were withdrawn every hour, measured spectrophotometrically at 230 nm and then returned to the system. Released DTX was determined by absorbance interpolation in a PBS calibration curve.

### Cell Culture

LnCaP and PC3 human PCa and C6 and U87 glioblastoma cell lines were obtained from ATCC (Manassas, VA, USA). The cells were grown in DMEM supplemented with 2 mM l-glutamine, 20 units/mL penicillin, 5 μg/mL streptomycin and 10% heat-inactivated fetal calf serum (Gibco, Whaltman, MA, USA). Cells were maintained at 37°C in a saturated humidity atmosphere containing 95% air and 5% CO_2_.

### Lactate Dehydrogenase Assay

Lactate dehydrogenase (LDH) toxicity assays were performed by measuring the release of LDH to the culture medium using the CytoTox96^®^ Non-Radioactive Cytotoxicity Assay kit (Promega, Madison, WI, USA) as previously described (Posadas et al., 2009). Briefly, tumoral cells were incubated for 72 h with free DTX (ranging from 0.1 to 1 μM) or different heterodimer formulations (nanocapsules or nanospheres), at increasing concentrations, loaded or not with DTX. The final nanoparticle concentration was adjusted to obtain the loaded DTX concentration that matched the free DTX concentration used in control experiments (0.1 to 1 μM). After treatment, the supernatants were collected and the intact cells were lysed using 0.1% (w/v) Triton X-100 in (0.9%) NaCl. Both the LDH released to culture media, as well as the LDH content within the cells, were determined spectrophotometrically at 490 nm on a 96-well plate reader (Infinite 200, Tecan, Salzburg, Austria) following the manufacturer’s instructions. LDH release was defined by the ratio LDH released/total LDH present in the cells, with the total LDH being 100%. All the samples were run in quadruplicate.

### Statistical Analysis

The non-parametric variance analysis (Kruskal–Wallis), followed by Dunn’s test, were used to evaluate statistical differences between groups. *P* < 0.05 was considered statistically significant. Statistical analyses were conducted using SPSS 13.0 (SPSS, Chicago, IL, USA).

## Results

### Preparation and Characterization of Self-Assembled Nanospheres and Nanocapsules from Giant Surfactants **1** and **2**

The interfacial solvent displacement method (Méndez-Ardoy et al., 2012) was used to engineer self-assembled nanospheres and nanocapsules from the CA_4_-βCD macromolecular constructs **1** and **2**. Dispersion of methanol solutions of the heterodimers into water containing a non-ionic hydrophilic surfactant (*Polysorbate 80*) instantaneously formed blank (unloaded) nanospheres. Evaporation of the organic solvent under reduced pressure allowed obtaining the nanoparticle suspension in water. For nanocapsule elaboration, pharmaceutically approved synthetic triglycerides (*Labrafac Lipophile WL1349*) were added to the organic phase containing the giant surfactant. Then, the solution was dropped into the aqueous phase to produce blank nanocapsules. Docetaxel-loaded NSs and NCs were prepared in a similar manner by co-formulation of the heterodimers and the drug (1:3 molar ratio) in the methanol solution followed by nanoprecipitation. The encapsulation efficiencies thus obtained ranked from 89% (for **1**-NS) and 83% (for **2**-NS) to 99% (for **1**-NC) and 98% (for **2**-NC), which are among the highest reported for docetaxel nanoformulations (Youm et al., 2011; Lollo et al., 2014). Most interestingly, the loading capacities (entrapped DXT/total amount of giant amphiphile and entrapped DTX, w/w, x100) were outstandingly high: 73% for **1**-NS, 71% for **2**-NS, 75% for **1**-NC and 74% for **2**-NC. The higher encapsulation efficiencies and loading capacities of nanocapsules, for a given giant surfactant entity, is consistent with the higher affinity of the strongly hydrophobic drug for the lipidic phase (triglycerides) at the inner core of these formulations. In any case, these values compare very favorably with data reported for other DXT-encapsulating systems, which typically exhibit loading capacities between 5 and 15% (Elsabahy et al., 2007; Hwang et al., 2008; Qi et al., 2013; Conte et al., 2016). It is worth noting that the mass ratio of DTX in the new nanospheres and the nanocapsules with respect to the total amount of excipients in the formulations (MNP **1** or **2** plus surfactant in the case of the NSs; MNP **1** or **2** plus oil plus surfactants in the case of NCs) is very close to 1 (1:1.1 for **1**-NS, 1:1.2 for **2**-NS, 1.0:1.02 for **1**-NC and 1.16 for **2**-NC), over one-order-of-magnitude lower than the 1:26 DTX:Polysorbate 80 mass ratio in the DTX commercial formulation Taxotere^®^.

The colloidal dispersions of blank or DTX-loaded NSs and NCs remained stable for more than 30 days both at room temperature and at 37°C. Dynamic light scattering (DLS) showed unimodal distributions of nanoparticle sizes, which remained unaltered for the whole 30-day’s period, with a small polydispersity index and negative ζ-potential in all cases (**Table 1**). Results are given as volume distribution of the major population by the mean diameter with its standard deviation. No significant differences were encountered when the data were expressed in intensity, volume or number distributions. This is consistent with a spherical topology of the nanoparticles. For blank nanospheres and nanocapsules, the hydrodynamic diameters ranged from 120 to 189 nm. When loaded with docetaxel, nanospheres decreased their hydrodynamic size to 20–35 nm (**Table 1**). In contrast, DTX-loaded nanocapsules exhibited hydrodynamic diameters in the range 200–265 nm, slightly higher as compared with the blank nanocapsules. Both NSs and NCs experienced a significant increase in ζ-potential upon loading with DTX, from about -35 to -15 mV, strongly suggesting that the drug locates in part at the nanoparticle surface, probably in the βCD cavities.

**Table 1:** Hydrodynamic diameter (nm), polydispersity index (PI) and **ζ**-Potential (mV) of blank and DTX-loaded nanospheres (NS) and nanocapsules (NC) prepared from **1** and **2**.

Formulation	Size (nm)	PI	ζ-Potential (mV)
1 blank NS	129 ± 1	0.04 ± 0.03	-31 ± 2
1 blank NC	120 ± 1	0.22 ± 0.01	-37.0 ± 1.6
2 blank NS	189 ± 1	0.05 ± 0.01	-34.2 ± 0.1
2 blank NC	152 ± 1	0.26 ± 0.02	-35.0 ± 0.9
1 DTX-NS	35 ± 1	0.28 ± 0.02	-13.3 ± 0.7
1 DTX-NC	267 ± 2	0.17 ± 0.02	-16.4 ± 0.9
2 DTX-NS	19.8 ± 0.3	0.23 ± 0.01	-12.7 ± 0.4
2 DTX-NC	209 ± 4	0.15 ± 0.01	-19.9 ± 0.8

*Data show mean ± SD of three independent determinations.*

**FIGURE 2:**
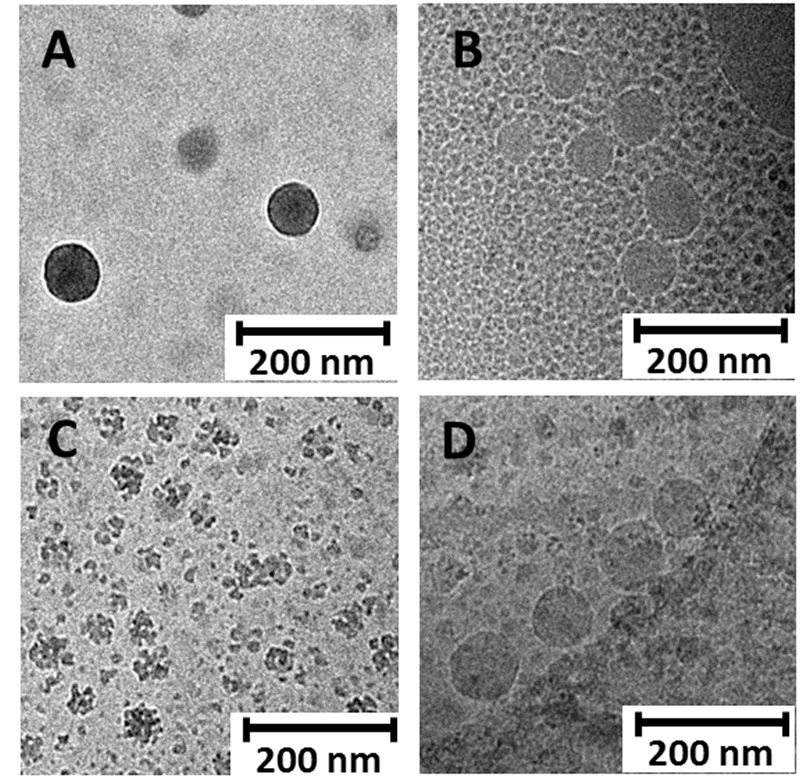
**Representative cryo-TEM images of unloaded nanospheres (A)** and nanocapsules **(B)** as well as docetaxel-loaded nanospheres **(C)** and nanocapsules **(D)** prepared from heterodimer **1**. In the absence of DTX, NS and NC of similar sizes (about 80 nm diameter) are observed **(A,B)**. After DTX-loading, NS size drastically decrease and entities of about 15 nm become observable, which eventually associate to give pomegranate-like aggregates **(C)**. On the contrary, NC size increases after DTX loading to about 150 nm diameter **(D)**. Morphology changes after DTX loading are consistent with alteration of the surface properties of the nanosystems, especially for NS, due to the presence of encapsulated DTX molecules in the peripheral βCD cavities.

Cryo-transmission electron microscopy (cryo-TEM) micrographs of the blank and DTX-loaded NSs and NCs evidenced quasi-spherical morphologies in all cases (**Figure 2**). Interestingly, the images reproduce the effect of DTX-loading on the size of the self-assembled nanoparticles already observed by DLS, that is, a moderate increase in the case of the nanocapsules (from about 80 to about 150 nm diameter; **Figure 2D** vs. **Figure 2B**), and a very significant decrease in the case of the NSs (from about 80 to about 15 nm; **Figure 2C** vs. **Figure 2A**). Most probably, blank nanospheres are formed by the aggregation of smaller entities, in agreement with previous atom force microscopy (AFM) observations. This hierarchical aggregation process is probably driven by hydrogen bonding interactions involving the βCD moieties at the surface of the NSs. It is interesting to speculate that inclusion of DTX molecules in the CD cavities of the loaded NSs weakens such interactions and stabilize the smaller species. Some pomegranate-like aggregates with varied sizes (30–60 nm) and morphologies, resulting from association of the elemental 15 nm nanoparticles, can nevertheless be observed in the micrographs of the DTX-loaded NSs (**Figure 2C**), which is probably the reason of the increased polydispersity of loaded as compared with blank NSs determined by DLS (**Table 1**). This behavior can be rationalized considering that the docetaxel molecules at the external shell can bridge βCD moieties at the surface of different NSs, since DTX possesses two aromatic rings fitting well in the βCD cavity. The ability of DTX to cluster molecular βCD derivatives has been previously documented (Benito et al., 2004).

### Docetaxel Release from CA_4_-βCD Nanospheres and Nanocapsules

**FIGURE 3:**
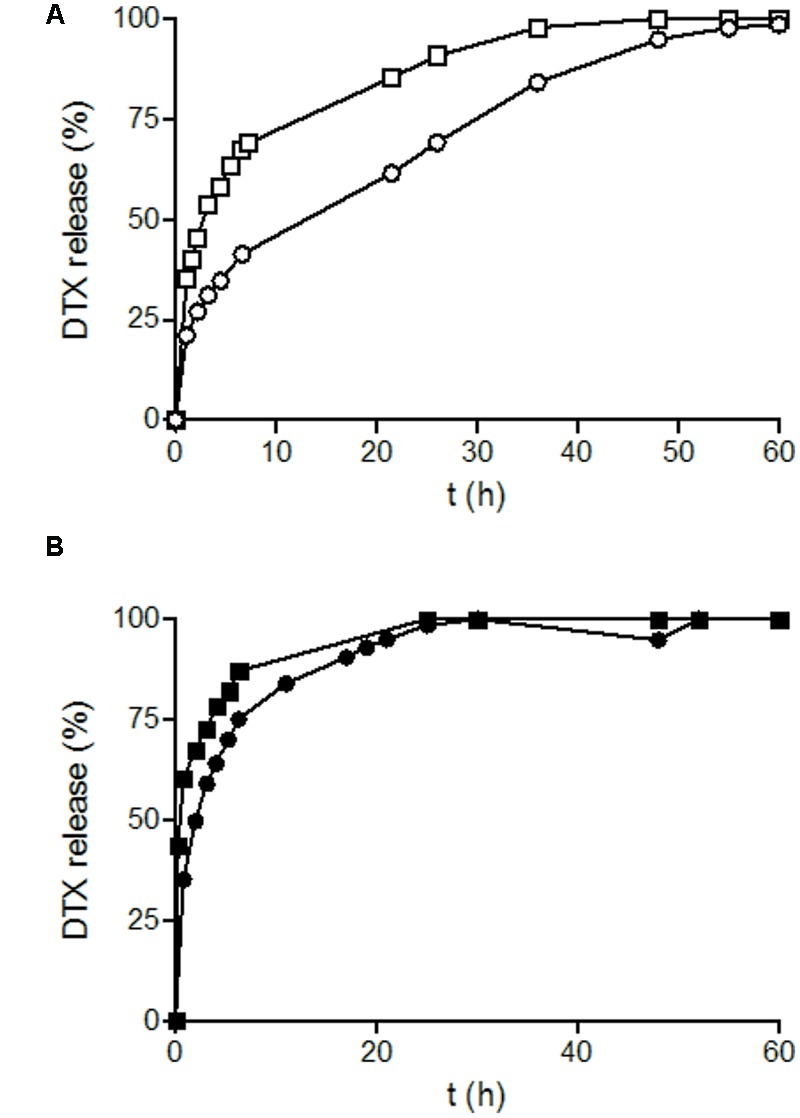
**Release profiles of docetaxel (DXT) as a function of time.** The figure represents DTX release profile from. **(A)** DTX-loaded heterodimer **1** nanocapsules (○); or nanospheres (□) and **(B)** DTX-loaded heterodimer **2** nanocapsules (●) or nanospheres (■). Experiments were performed in PBS (pH 7.4) at 37°C.

The docetaxel release kinetics followed a hyperbolic profile both for the DTX-NS and DTX-NC formulations, with an initial burst (6–8 h) followed by a sustained delivery over 30–60 h (**Figure 3**). The nanoparticles obtained from giant surfactant **1**, containing four hexyl chains at the calix[4]arene MNP module, exhibited significantly longer DTX release times as compared with formulations prepared from **2**, which instead have four dodecyl chains at the CA_4_ component. This result is consistent with the strong dependence of the properties of nanostructured materials assembled from giant molecules on chemical modifications in the MNP components (Huang et al., 2015).

Given the aforementioned very significant changes in the surface properties of the NSs and the NCs upon loading with DTX, it seems reasonable to speculate that the fast-releasing DTX fraction corresponds to the drug hosted in the outer βCD shell, whereas the slow-releasing portion accounts for the drug encapsulated in the hydrophobic CA_4_-encircled core. In the case of the NCs, the fast-releasing fraction represents about 60% of the total drug, independently of the giant amphiphile structure (**1** or **2**). However, the measured DTX-loading capabilities indicate an average 2.5:1 molar DTX:giant amphiphile ratio for **1**-NS and **2**-NS, meaning that the maximum theoretical fraction of the drug hosted in βCD cavities (supposing full occupation of the βCD cavities and a 1:1 DTX:βCD stoichiometry) is 40%. The results can be rationalized assuming that part of the fast-releasing DTX fraction is indeed hosted in the intermediate region defined by the spacer linking the βCD and CA_4_ modules, which remains relatively flexible and open. In the case of the NCs, the fast releasing DTX fraction is much smaller than in the case of the NSs, about 25% of the total drug. This is in agreement with the proportionally much lower volume of the βCD shell in the NC constructs, where the giant amphiphile molecules are presumed to form a monolayer coating the oily nanodrops.

### Cytotoxic Action of DTX-Loaded Nanoparticles

**FIGURE 4:**
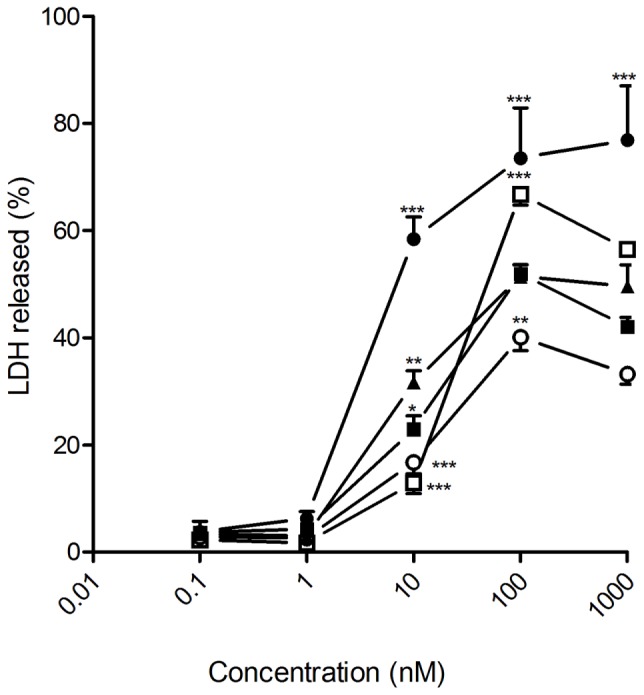
**Antitumoral effect of free and encapsulated docetaxel on LnCaP human prostate cancer (PCa) cells.** LnCaP cancer cells were exposed to increasing concentrations of either free DTX (▲) or docetaxel encapsulated in heterodimer 1 nanocapsules (○); heterodimer **1** nanospheres (□); heterodimer **2** nanocapsules (●) or heterodimer **2** nanospheres (■) for 72 h. LDH release to the medium was considered an index of cellular death. Data represent mean ± SEM of 10 to 18 cells. ^∗^*p* < 0.05, ^∗∗^*p* < 0.01, ^∗∗∗^*p* < 0.001 when compared to free DTX.

**FIGURE 5:**
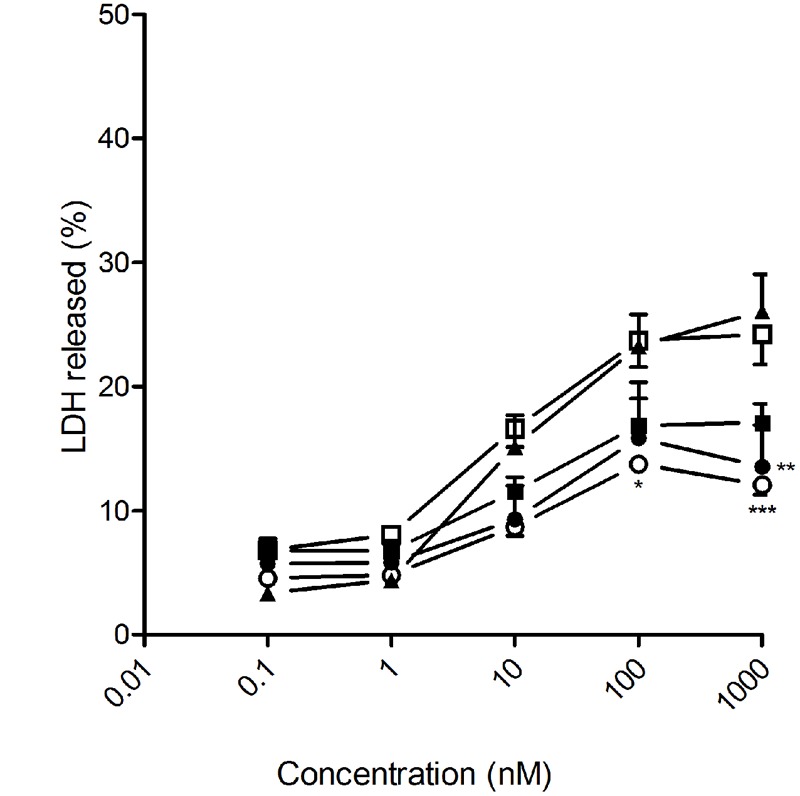
**Antitumoral effect of free and encapsulated docetaxel on PC3 human PCa cells.** PC3 cancer cells were exposed to increasing concentrations of either free DTX (▲) or docetaxel encapsulated in heterodimer **1** nanocapsules (○); heterodimer **1** nanospheres (□); heterodimer **2** nanocapsules (●)or heterodimer **2** nanospheres (■) for 72 h. LDH release to the medium was considered an index of cellular death. Data represent mean ± SEM of 16 to 44 cells. ^∗^*p* < 0.05, ^∗∗^*p* < 0.01, ^∗∗∗^*p* < 0.001 when compared to free DTX.

The cytotoxic effect of DTX-loaded NSs and NCs, prepared from the βCD-CA_4_ amphiphilic heterodimers **1** and **2**, was tested on prostate and glioblastoma tumoral cell lines by exposing them to increasing concentrations of the different nanoparticle formulations for 72 h. Total DTX concentration encapsulated in the nanoparticles ranged from 1 nM to 1 μM. As a positive control, the same cells were exposed to free DTX (13% ethanol solution) at the same concentrations. DTX is one of the drugs used in the first-line treatment of PCa. In this work we tested the effects of the giant surfactant-based DTX formulations on two human PCa cell lines: LnCaP, which is hormone-sensitive and represents an early stage of PCa, and PC3, which is more resistant to chemotherapy and is a model for hormone-refractory PCa. As shown in **Figure 4**, DTX-loaded nanocapsules engineered from macromolecular heterodimer **2** were significantly more efficient than free DTX at inducing LnCaP tumoral cell death at concentrations above 1 nM. The DTX-loaded nanospheres obtained from heterodimers **1** and **2** were comparatively less effective, but exhibited an efficacy similar to that of free DTX. Regarding PC3 cells, DTX-loaded nanocapsules formulated with heterodimer **1** showed a cytotoxic effect comparable to free DTX, with the other formulations being less effective than free DTX (**Figure 5**). It is important to note that the toxicity of free DTX in PC3 cells is reduced in comparison to that observed for LnCaP cells. This is consistent with the higher resistance to DTX observed in hormone-independent advanced PCa.

**FIGURE 6:**
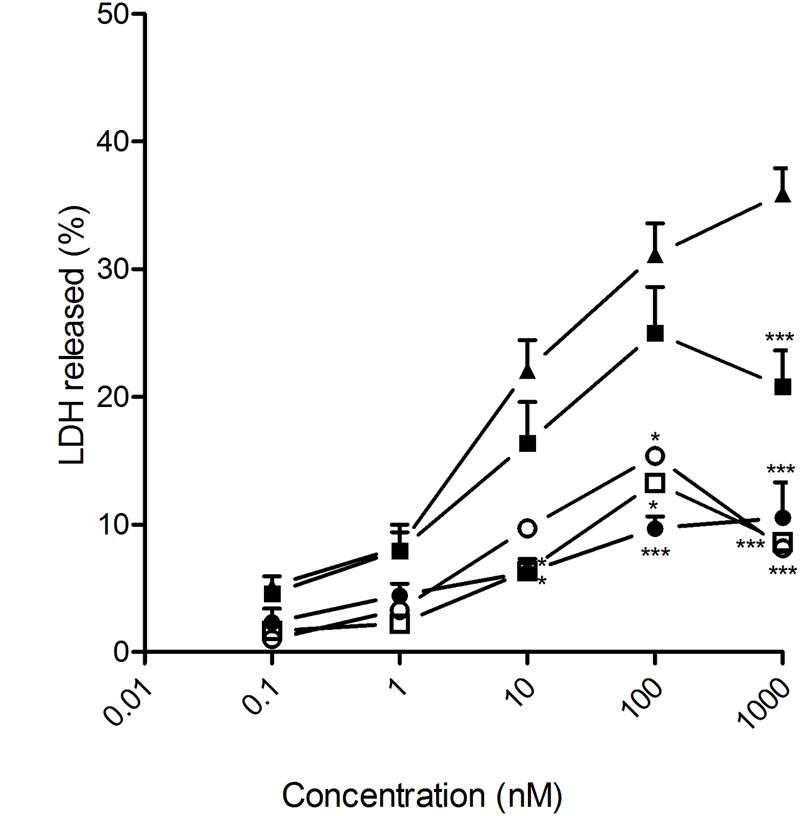
**Antitumoral effect of free and encapsulated docetaxel on U87 human glioblastoma cancer cells.** U87 cancer cells were exposed to increasing concentrations of either free DTX (▲) or docetaxel encapsulated in heterodimer **1** nanocapsules (○); heterodimer **1** nanospheres (□); heterodimer **2** nanocapsules (●) or heterodimer **2** nanospheres (■) for 72 h. LDH release to the medium was considered an index of cellular death. Data represent mean ± SEM of 16 to 22 cells. ^∗^*p* < 0.05, ^∗∗^*p* < 0.01, ^∗∗∗^*p* < 0.001 when compared to free DTX.

**FIGURE 7:**
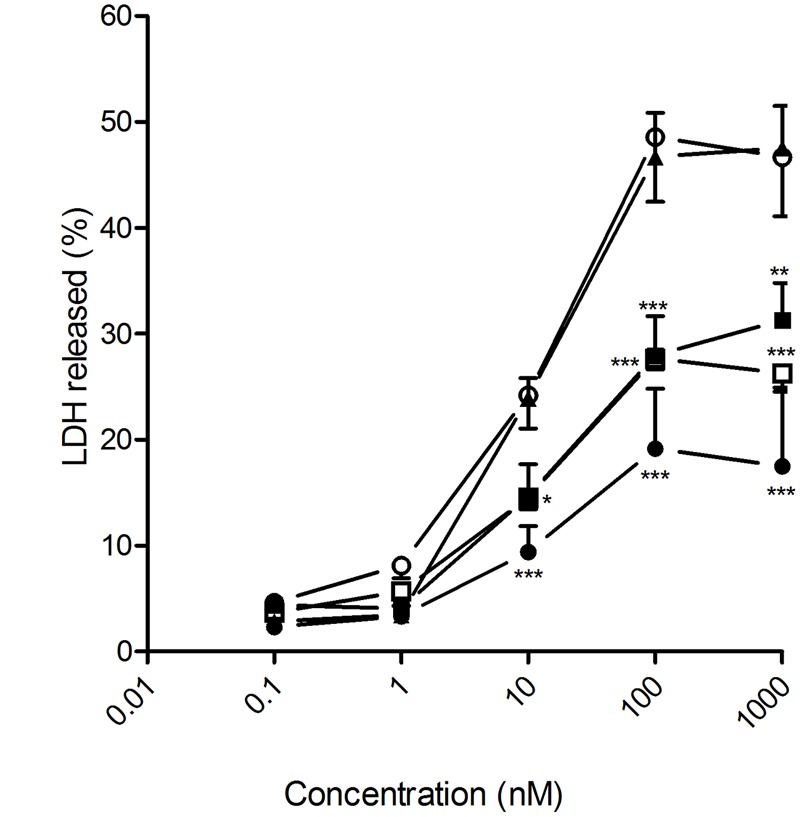
**Antitumoral effect of free and encapsulated docetaxel on rat glioblastoma C6 cancer cells.** C6 cancer cells were exposed to increasing concentrations of either free DTX (▲) or docetaxel encapsulated in heterodimer **1** nanocapsules (○); heterodimer **1** nanospheres (□); heterodimer **2** nanocapsules (●) or heterodimer **2** nanospheres (■) for 72 h. LDH release to the medium was considered an index of cellular death. Data represent mean ± SEM of 20 to 32 cells. ^∗^*p* < 0.05, ^∗∗^*p* < 0.01, ^∗∗∗^*p* < 0.001when compared to free DTX.

Docetaxel has been also used to treat metastasized glioblastoma (Astner et al., 2006). In this sense, the different DTX-loaded nanoparticles prepared in this work induced human glioblastoma U87 cell death in 72 h. DTX-loaded nanospheres prepared from heterodimer **2**, which exhibits the fastest DTX release profile of all four assayed nanoformulations, proved the most efficacious, inducing tumoral cell death levels analogous to those obtained by the ethanolic free DTX at 100-nM concentration (**Figure 6**). In stark contrast, DTX-loaded nanocapsules formulated from heterodimer **1**, which shows the slowest release kinetics, turned out to be the most efficient in rat glioblastoma C6 cells, which offer a widely used murine model for human glioblastoma (**Figure 7**). No toxicity was observed for the blank nanoparticles in any of the four cell lines studied (data not shown).

## Discussion

In this work we have formulated self-assembled nanoparticles from giant surfactants composed of two covalently connected MNP entities; namely, a hydrophilic β-cyclodextrin and a hydrophobic calix[4]arene module. Our purpose was twofold: (a) to probe the suitability of the heterodimeric CA_4_-βCD prototype as a versatile platform to optimize drug encapsulation, delivery and release for a given cell type by finely tuning the primary macromolecular structure and/or the architecture of the nanoassembly; and (b) to develop new DTX formulations with increased water solubility and bioavailability, without the need for a co-solvent, thus retaining full anticancer efficacy. As a proof of concept, we have engineered a short library of four nanosystems, two having nanosphere-type topology and two with a nanocapsule-type arrangement, from the CA_4_-βCD heterodimers **1** and **2**. All the NS and NC constructs were characterized before and after loading with DTX and their antitumoral action was evaluated in two prostatic cancer and two glioblastoma cell lines and compared with a free DTX formulation, containing 13% ethanol and Polysorbate 80, currently used in hospitals (Taxotere^®^).

The CA_4_-βCD giant surfactants **1** and **2** central to this work were efficiently obtained by high-yielding “click”-type thiourea coupling reactions between the isothiocyanate-armed tetraalkylated CA_4_ derivative **3** or **4**, which provides the hydrophobic MNP component in the final amphipathic macromolecular adduct, and the amine-equipped βCD derivative **5**, which supplies the hydrophilic MNP module (**Figure 1**). We must bear in mind that the calix[4]arene scaffold, in its cone conformation, offers an excellent platform for tight compaction of hydrophobic tails located at its narrower rim, which enhances the giant surfactant system self-assembling capabilities in an aqueous environment (Gallego-Yerga et al., 2015b). In the resulting nanoaggregates, the CA_4_ moieties will be located in the core, providing a hydrophobic matrix that is expected to be well-suited to accommodate hydrophobic drugs. The drug loading capacity in the nanoparticle interior and the release kinetics will depend on the architecture of the assembly (NS or NC) and on the nature of the hydrophobic tails installed at the phenolic oxygen atoms (hexyl for **1** and dodecyl for **2**). The β-cyclodextrin component will remain instead at the external shell of the nanosystems, in contact with the bulk medium. The βCD cavities will be then available for encapsulating either additional drug or a third species, which can be exploited to program different drug release profiles or to modify the nanoparticle surface, e.g., by supramolecular incorporation of functional elements for targeting purposes. It should be emphasized that contrary to other giant amphiphiles based in block copolymers (Wilks et al., 2013) thereby intrinsically polydisperse, the CA_4_-βCD giant surfactants considered in this work are single isomers with perfectly defined molecular structure and obtained in genuine monodisperse form. Differences in their self-assembling properties can be thus directly related to structural differences at the atomic level.

The two giant surfactants **1** and **2** allowed accessing either nanospheres or nanocapsules after nanoprecipitation by dropping a methanol solution into a water phase, depending on whether or not the organic solvent additionally incorporates a non-ionic hydrophilic surfactant or pharmaceutically approved synthetic triglycerides, respectively. DTX was efficiently integrated into the NS and NC formulations by co-dissolving with **1** or **2** prior to nanoprecipitation. Indeed, drug loading capabilities higher that 75% were achieved in all cases, which rank among the highest reported for DTX nanocarriers.

Drug loading had a significant impact on the nanoassembly properties. Thus, the size of the nanospheres was very significantly decreased after DTX incorporation, whereas that of the nanocapsules experienced only a slight increase (**Figure 2**). Probably, inclusion of a fraction of the drug in the cavities of the βCD modules affects their capacity for hydrogen-bonding interplay, preventing aggregation phenomena. Indeed, the cryo-TEM micrographs of the DTX loaded nanospheres formulated with **1** (**Figure 2C**) show the presence of small (15 nm) spherical entities that probably engage in hierarchical self-assembling in the absence of the drug, affording higher (about 100 nm) nanoparticles (**Figure 2A**). This observation is in full accordance with previous atom force microscopy data (Gallego-Yerga et al., 2014).

Hydrodynamic diameters determined by DLS were overall consistent with cryo-TEM data, considering the different operational principles of both techniques (Geze et al., 2004). DTX-loading also had a significant impact on the surface charge of the nanoparticles. The ζ potential increased by about 20 mV when formulated with the drug as compared with blank NSs or NCs (from about -35 to -15 mV), strongly supporting that the drug locates in part at the external shell, probably after partial inclusion in the cavities of the βCD moieties. Indeed, it has been previously shown that DTX can form inclusion complexes with βCD derivatives in which an aromatic ring of the DTX molecule is hosted inside the βCD cavity (Benito et al., 2004). The stability of these complexes is enhanced if the two phenyl moieties in DTX can interact simultaneously with two βCD units, which is likely the case at the surface of the CA_4_-βCD assemblies. The capacity of DTX to bridge βCD units can also explain the presence of pomegranate-like aggregates in the DTX-loaded NSs revealed by cryo-TEM (**Figure 2C**), which correlates with the higher polydispersity index determined by DLS as compared to blank NSs. The ability of the nanoaggregates to retain DTX at the hydrophilic corona through inclusion phenomena is probably further enhanced by the multivalent presentation of βCD motifs, which likely favors sliding and rebinding processes of the drug. Both the nanospheres and the nanocapsules thus behave as multicavity systems with at least two functional regions: the core and the shell. A fraction of the loaded DTX will be encapsulated in the inner core, relatively isolated from the environment, and another fraction will stay at the shell and be partially exposed to the medium.

The profiles of DTX release kinetics from DTX-NS and DTX-NC formulations are overall compatible with the core-shell architecture of the assemblies and the proposed differential distribution of the stored drug between these two distinct compartmented regions. Thus, the curves reveal an initial fast release period that likely accounts for the exchange of the DTX molecules included in βCD cavities or entrapped in the region defined by the segment connecting the βCD and CA_4_ modules. If this assumption is correct, the DTX fraction hosted in the shell would account for about 60% in the case of the nanospheres and 25% in the case of the nanocapsules.

Diffusion of the drug out of the nanoassembly core is a slower process and is probably responsible for the sustained release period. The presence of the lipidic phase in the core of nanocapsules leads to a delayed DTX release as compared with the corresponding nanosphere formulations. Most notably, DTX release was found to be significantly faster from DTX-loaded nanospheres and nanocapsules assembled from giant surfactant **2** as compared with the analogous formulations prepared from the homologous CA_4_-βCD heterodimer **1**, even though no relevant differences were observed in the nanoparticle sizes, topologies or ζ-potential. It is possible that the higher conformational flexibility of dodecyl over hexyl tails translates into less compact arrangements after self-assembly, facilitating the exchange between the nanoparticle core and the bulk aqueous exterior. The interplay of molecular (hexyl or decyl chains at the CA_4_ moiety) and nanostructure features (NS or NC arrangement) results in a gradation of the DTX release rate that follows the order **2** DTX-NS>**1** DTX-NS≈**2** DTX-NC>**1** DTX-NC. This result highlights the unique possibilities for finely programming the properties of nanometric devices assembled from giant surfactants through the controlled chemical modification of a single individual MNP component. Interestingly, all NSs and NCs prepared in this work, either blank or DTX-loaded, remained stable as colloidal solutions at pH 7 for over 30 days at 25 or 37°C, as inferred from the absence of any precipitate and the virtually identical DLS and cryo-TEM results recorded over this time period. The DTX release kinetics from the drug-loaded nanosystems were also virtually identical after storage, meaning that the relatively fast release observed for NSs must be ascribed to the particular characteristics of this self-assembled arrangement and not to stability problems.

The main drawback of current commercial DTX formulations is their low water solubility. This requires dilution with ethanol before administration of the drug, which exacerbates the already high intrinsic DTX toxicity (Zhang and Zhang, 2013). The giant amphiphile-based nanosphere and nanocapsule formulations studied here are all able to efficiently incorporate DTX, solubilize it in water without the need for a co-solvent and fully release the drug payload in a biological environment over a 30 to 60 h period, yielding effective DTX concentrations that are able to induce tumoral cancer cell death. The vials obtained after the formulation procedure described in Methods contained 950 mg of DTX and about the same amount of excipients (including the giant amphiphile **1** or **2**, surfactants and, in the case of NCs, the oil component) in 1 mL of plain water, that is a mass ratio of drug to excipient very close to unity. For comparison, the commercial formulation of DTX (Taxotere^®^) contains 40 mg of DTX and 1040 mg of Polysorbate 80 in 1 mL of 13% aqueous ethanol, meaning a mass ratio of drug to excipient of 1:26. Another important advantage of the new NS and NC formulations is that they remained stable for periods over 30 days at room temperature, whereas Taxotere must be formulated from a solution of anhydrous DTX in Polysorbate 80 and used in 8 h due to drug instability in the hydroethanolic medium.

Most of the cell lines tested for sensitivity to DTX-induced cell death had IC_50_ values between 5 and 50 nM (Clarke and Rivory, 1999). This was also the case for the four cell lines considered in this study (LnCaP, PC3, U87 and C6) when treated with the ethanol-diluted DTX formulation, as well as with the different DTX-loaded nanoparticles. We therefore focused on the range of 10 to 100 nM total DTX concentration to compare the effectiveness of free versus encapsulated DTX formulations and to analyze the potential advantages of encapsulating the drug in the giant surfactant-based nanocarriers. In all cases, the corresponding blank nanospheres or nanocapsules prepared from **1** or **2** at identical concentration, used as controls in parallel assays, did not promote any cytotoxicity. The anticancer effectiveness of the formulations was found to be highly dependent on the cell linage, emphasizing the need for approaches that permit optimization of the carrier on a case-by-case basis. Giant surfactants are particularly well-suited for that purpose. Thus, DTX-loaded nanocapsules were more effective than the corresponding nanospheres in human LnCap cells, which points to bigger nanoparticles favoring the anticancer activity in this particular cell type. For a given nanocarrier configuration (NS or NC), the efficiency at promoting LnCap cell death increased with the drug release rate, with the optimal DTX-NC formulation prepared from heterodimer **2** exhibiting a much higher efficiency than the commercial free DTX formulation in the 10 to 100 nM range. In stark contrast, the hormone-resistant human PCa PC3 cell line was more sensitive to small size DTX-loaded nanospheres than to the corresponding nanocapsules. The DTX-NS formulation assembled from heterodimer **1**, which has a significantly slower release profile than the analogous DTX-NS formulation prepared from **2**, yielded the best results of the four systems tested, showing a similar cytotoxic effect to the free DTX formulation. In the case of the human glioma U87 cell line, the DTX-loaded nanospheres engineered from the CA_4_-βCD heterodimer **2**, which combine small size and fast drug release, were significantly more efficacious than the other three nanoformulations assayed, whereas the rat glioblastoma cell line C6 was instead more sensitive to the higher size and slow DTX releasing nanocapsules assembled from **1**. In both cases the optimal system behaved as efficiently as the free DTX formulation in the same experimental setting.

The differences observed in the sensitivity of the different cell lines to the DTX-loaded nanoparticles might reflect differences in the ability of the nanosystems to cross the plasma membrane of each cell type and dissimilarities in their susceptibility to either a slow or fast drug release profile. Treating human PCa LnCaP cell line with DTX-loaded nanocapsules prepared from heterodimer **2** represents a particularly favorable scenario, with the giant surfactant-based nanoformulation being markedly superior at inducing cell death as compared with the free DTX ethanolic formulation. Most importantly, by properly choosing the giant surfactant precursor (**1** or **2**) and the optimal nanoassembly configuration (NS or NC), it was possible to find a nanoformulation for every cell type that performed at least as efficiently as free DTX, ensuring full water solubility without the need for any co-solvent. The core-shell architecture of the nanocarriers, which exposes the βCD component of the CA_4_-βCD heterodimeric building blocks to the bulk, further opens the door to the possibility of supramolecular decoration of the NS or NC surface with targeting groups for site specific DTX delivery to tumoral tissues (Yin et al., 2013). Encapsulation of the drug is additionally expected to improve its bioavailability and decrease the variability of the treatment by avoiding premature clearance through biological fluids. The docetaxel-mediated clinical effect and its hematologic toxicity have been found to correlate very well with the free drug concentration that is not bound to serum proteins (Baker et al., 2005), which amounts to only 5% of the total drug concentration in plasma. The remaining 95% of the drug is bound mainly to albumin and α1-acid glycoprotein (AAG). The large disparity in AAG levels among different patients is largely responsible for the observed discrepancies in DTX effectiveness and toxicity (Bruno et al., 1998). Carbohydrate coating is an efficient strategy to prevent serum protein binding to nanoparticles, similarly to polyethylene glycol “stealth” coating (Amoozgar and Yeo, 2012). The presence of glucose units at the outer surface of the nanosystems assembled from CA_4_-βCD heterodimers was indeed purposely intended to make the new DTX formulations stealth.

To the best of our knowledge, the body of results reported herein provides the first evidence of the potential of the “macromolecular precise synthesis” and “giant surfactant” concepts in strategies directed at optimizing drug nanoformulations. By covalently connecting β-cyclodextrin and calix[4]arene MNPs, heterodimers with perfectly controlled structures can be accessed. The self-assembly of the resulting CA_4_-βCD giant surfactants yields core-shell nanospheres or nanocapsules, depending on the nanoprecipitation protocol, with drug encapsulation and release properties that are closely related to the primary macromolecular architecture. The versatility of the prototype offers unprecedented opportunities to conduct drug and cell-oriented SAR studies, as exemplified by the set of data obtained on the anticancer activity of docetaxel-loaded nanoassemblies against pancreatic and glioblastoma cells. Giant amphiphile-based formulations surpassing or matching the antitumoral activity of the free DTX formulation were identified in all cases, overcoming the DTX water solubility problems. The proposed self-assembled nanocarriers are further intended to be biocompatible, to have a low propensity to undergo protein serum adsorption and to be compatible with the incorporation of targeting moieties for tumor-specific drug delivery, by virtue of exposing cyclooligosaccharidic βCD units at their surface (Mogosanu et al., 2016). This should allow them to bypass healthy cells, thus reducing the side-effects associated with DTX treatment. We are continuing to work along these lines in our laboratories.

## Author Contributions

LG-Y, IP, CT, and JR-A made substantial contributions to acquisition and analysis of the data. FS, COM, AC, JMGF and VC designed the work and contributed to the interpretation of the data. LG-Y, CT, COM, JMGF, and VC drafted the work. LG-Y, IP, CT, JR-A, FS, and AC revised the draft for important intellectual content. All authors approved the final version of the manuscript to be submitted. All authors agreed to be accountable for all aspects of the work.

## Conflict of Interest Statement

The authors declare that the research was conducted in the absence of any commercial or financial relationships that could be construed as a potential conflict of interest.

## References

[B1] Amoozgar Z. Yeo Y. (2012). Recent advances in stealth coating of nanoparticle drug delivery systems. *Wiley Interdiscip. Rev. Nanomed. Nanobiotechnol.* 4 219–233. 10.1002/wnan.1157 22231928 PMC3288878

[B2] Aranda C. Urbiola K. Méndez Ardoy A. Garcia Fernandez J. M. Ortiz Mellet C. de Ilarduya C. T. (2013). Targeted gene delivery by new folate-polycationic amphiphilic cyclodextrin-DNA nanocomplexes in vitro and in vivo. *Eur. J. Pharm. Biopharm.* 85 390–397. 10.1016/j.ejpb.2013.06.011 23811437

[B3] Astner S. T. Pihusch R. Nieder C. Rachinger W. Lohner H. Tonn J. C. (2006). Extensive local and systemic therapy in extraneural metastasized glioblastoma multiforme. *Anticancer Res.* 26 4917–4920. 17214362

[B4] Attili-Qadri S. Karra N. Nemirovski A. Schwob O. Talmon Y. Nassar T. (2013). Oral delivery system prolongs blood circulation of docetaxel nanocapsules via lymphatic absorption. *Proc. Natl. Acad. Sci. U.S.A.* 110 17498–17503. 10.1073/pnas.1313839110 24101508 PMC3808655

[B5] Baker S. D. Li J. ten Tije A. J. Figg W. D. Graveland W. Verweij J. (2005). Relationship of systemic exposure to unbound docetaxel and neutropenia. *Clin. Pharmacol. Ther.* 77 43–53. 10.1016/j.clpt.2004.09.005 15637530

[B6] Benito J. M. Gomez-Garcia M. Ortiz Mellet C. Baussanne I. Defaye J. Garcia Fernandez J. M. (2004). Optimizing saccharide-directed molecular delivery to biological receptors: design, synthesis, and biological evaluation of glycodendrimer-cyclodextrin conjugates. *J. Am. Chem. Soc.* 126 10355–10363. 10.1021/ja047864v 15315450

[B7] Bruno R. Hille D. Riva A. Vivier N. ten Bokkel Huinnink W. W. van Oosterom A. T. (1998). Population pharmacokinetics/pharmacodynamics of docetaxel in phase II studies in patients with cancer. *J. Clin. Oncol.* 16 187–196. 10.1200/JCO.1998.16.1.187 9440742

[B8] Cho H. J. Park J. W. Yoon I. S. Kim D. D. (2014). Surface-modified solid lipid nanoparticles for oral delivery of docetaxel: enhanced intestinal absorption and lymphatic uptake. *Int. J. Nanomed.* 9 495–504. 10.2147/IJN.S56648 PMC3894956 24531717

[B9] Clarke S. J. Rivory L. P. (1999). Clinical pharmacokinetics of docetaxel. *Clin. Pharmacokinet.* 36 99–114. 10.2165/00003088-199936020-00002 10092957

[B10] Conte C. Scala A. Siracusano G. Sortino G. Pennisi R. Piperno A. (2016). Nanoassemblies based on non-ionic amphiphilic cyclodextrin hosting Zn(II)-phthalocyanine and docetaxel: Design, physicochemical properties and intracellular effects. *Colloids Surf. B Biointerfaces* 146 590–597. 10.1016/j.colsurfb.2016.06.047 27424090

[B11] Dumontet C. Sikic B. I. (1999). Mechanisms of action of and resistance to antitubulin agents: microtubule dynamics, drug transport, and cell death. *J. Clin. Oncol.* 17 1061–1070. 10.1200/JCO.1999.17.3.1061 10071301

[B12] Elsabahy M. Perron M. E. Bertrand N. Yu G. E. Leroux J. C. (2007). Solubilization of docetaxel in poly(ethylene oxide)-block-poly(butylene/styrene oxide) micelles. *Biomacromolecules* 8 2250–2257. 10.1021/bm070226v 17579476

[B13] Francois A. Laroche A. Pinaud N. Salmon L. Ruiz J. Robert J. (2011). Encapsulation of docetaxel into PEGylated gold nanoparticles for vectorization to cancer cells. *Chem. Med. Chem.* 6 2003–2008. 10.1002/cmdc.201100311 21834092

[B14] Gajbhiye V. Jain N. K. (2011). The treatment of Glioblastoma Xenografts by surfactant conjugated dendritic nanoconjugates. *Biomaterials* 32 6213–6225. 10.1016/j.biomaterials.2011.04.057 21616528

[B15] Gallego-Yerga L. Blanco-Fernandez L. Urbiola K. Carmona T. Marcelo G. Benito J. M. (2015a). Host-guest-mediated DNA templation of polycationic supramolecules for hierarchical nanocondensation and the delivery of gene material. *Chem. Eur. J.* 21 12093–12104. 10.1002/chem.201501678 26184887

[B16] Gallego-Yerga L. Lomazzi M. Franceschi V. Sansone F. Ortiz Mellet C. Donofrio G. (2015b). Cyclodextrin- and calixarene-based polycationic amphiphiles as gene delivery systems: a structure-activity relationship study. *Org. Biomol. Chem.* 13 1708–1723. 10.1039/c4ob02204a 25474077

[B17] Gallego-Yerga L. Lomazzi M. Sansone F. Ortiz Mellet C. Casnati A. Garcia Fernandez J. M. (2014). Glycoligand-targeted core-shell nanospheres with tunable drug release profiles from calixarene-cyclodextrin heterodimers. *Chem. Commun.* 50 7440–7443. 10.1039/c4cc02703e 24875493

[B18] Geze A. Putaux J. L. Choisnard L. Jehan P. Wouessidjewe D. (2004). Long-term shelf stability of amphiphilic ß-cyclodextrin nanosphere suspensions monitored by dynamic light scattering and cryo-transmission electron microscopy. *J. Microencapsul.* 21 607–613. 10.1080/02652040400008457 15762318

[B19] Giannakakou P. Gussio R. Nogales E. Downing K. H. Zaharevitz D. Bollbuck B. (2000). A common pharmacophore for epothilone and taxanes: molecular basis for drug resistance conferred by tubulin mutations in human cancer cells. *Proc. Natl. Acad. Sci. U.S.A.* 97 2904–2909. 10.1073/pnas.040546297 10688884 PMC16028

[B20] Gottesman M. M. Fojo T. Bates S. E. (2002). Multidrug resistance in cancer: role of ATP-dependent transporters. *Nat. Rev. Cancer* 2 48–58. 10.1038/nrc706 11902585

[B21] He L. Yang C. P. Horwitz S. B. (2001). Mutations in beta-tubulin map to domains involved in regulation of microtubule stability in epothilone-resistant cell lines. *Mol. Cancer Ther.* 1 3–10. 12467233

[B22] Huang M. Hsu C. H. Wang J. Mei S. Dong X. Li Y. (2015). Self-assembly. Selective assemblies of giant tetrahedra via precisely controlled positional interactions. *Science* 348 424–428. 10.1126/science.aaa2421 25908818

[B23] Hwang H. Y. Kim I. S. Kwon I. C. Kim Y. H. (2008). Tumor targetability and antitumor effect of docetaxel-loaded hydrophobically modified glycol chitosan nanoparticles. *J. Control. Release* 128 23–31. 10.1016/j.jconrel.2008.02.003 18374444

[B24] James N. D. Sydes M. R. Clarke N. W. Mason M. D. Dearnaley D. P. Spears M. R. (2016). Addition of docetaxel, zoledronic acid, or both to first-line long-term hormone therapy in prostate cancer (STAMPEDE): survival results from an adaptive, multiarm, multistage, platform randomised controlled trial. *Lancet* 387 1163–1177. 10.1016/S0140-6736(15)01037-5 26719232 PMC4800035

[B25] Kaneda T. Fuyimoto T. Goto J. Asano K. Jasufuku J. H. J. Ozono C. (2002). New large-scale preparations of versatile 6-O-monotosyl and 6-monohydroxy permethylated α-, β-, and γ-cyclodextrins. *Chem. Lett.* 31 514–515. 10.1246/cl.2002.514

[B26] Keldermann E. Derhaeg L. Heesink G. J. T. Verboom W. Engbersen J. F. J. Van Hulst N. F. (1992). Nitrocalix[4]arenes as molecules for second-order nonlinear optics. *Angew. Chem. Int. Ed. Engl.* 31 1075–1077. 10.1002/anie.199210751

[B27] Kurkov S. V. Loftsson T. (2013). Cyclodextrins. *Int. J. Pharm.* 453 167–180. 10.1016/j.ijpharm.2012.06.055 22771733

[B28] Lee E. Kim H. Lee I. H. Jon S. (2009). In vivo antitumor effects of chitosan-conjugated docetaxel after oral administration. *J. Control. Release* 140 79–85. 10.1016/j.jconrel.2009.08.014 19712714

[B29] Lollo G. Rivera-Rodriguez G. R. Bejaud J. Montier T. Passirani C. Benoit J. P. (2014). Polyglutamic acid-PEG nanocapsules as long circulating carriers for the delivery of docetaxel. *Eur. J. Pharm. Biopharm.* 87 47–54. 10.1016/j.ejpb.2014.02.004 24530693

[B30] Méndez-Ardoy A. Gómez-García M. Geze A. Putaux J. L. Wouessidjewe D. Ortiz Mellet C. (2012). Monodisperse nanoparticles from self-assembling amphiphilic cyclodextrins: modulable tools for the encapsulation and controlled release of pharmaceuticals. *Med. Chem.* 8 524–532. 10.2174/157340612801216265 22571191

[B31] Mendez-Ardoy A. Urbiola K. Aranda C. Ortiz Mellet C. Garcia-Fernandez J. M. de Ilarduya C. T. (2011). Polycationic amphiphilic cyclodextrin-based nanoparticles for therapeutic gene delivery. *Nanomedicine* 6 1697–1707. 10.2217/nnm.11.59 22122582

[B32] Mogosanu G. D. Grumezescu A. M. Bejenaru C. Bejenaru L. E. (2016). Polymeric protective agents for nanoparticles in drug delivery and targeting. *Int. J. Pharm.* 510 419–429. 10.1016/j.ijpharm.2016.03.014 26972379

[B33] Montero A. Fossella F. Hortobagyi G. Valero V. (2005). Docetaxel for treatment of solid tumours: a systematic review of clinical data. *Lancet Oncol.* 6 229–239. 10.1016/S1470-2045(05)70094-2 15811618

[B34] Posadas I. Lopez-Hernandez B. Clemente M. I. Jimenez J. L. Ortega P. de la M. J. (2009). Highly efficient transfection of rat cortical neurons using carbosilane dendrimers unveils a neuroprotective role for HIF-1alpha in early chemical hypoxia-mediated neurotoxicity. *Pharm. Res.* 26 1181–1191. 10.1007/s11095-009-9839-9 19191011

[B35] Qi C. Zhu Y. J. Zhao X. Y. Lu B. Q. Tang Q. L. Zhao J. (2013). Highly stable amorphous calcium phosphate porous nanospheres: microwave-assisted rapid synthesis using ATP as phosphorus source and stabilizer, and their application in anticancer drug delivery. *Chem. Eur. J.* 19 981–987. 10.1002/chem.201202829 23180605

[B36] Rao B. M. Chakraborty A. Srinivasu M. K. Devi M. L. Kumar P. R. Chandrasekhar K. B. (2006). A stability-indicating HPLC assay method for docetaxel. *J. Pharm. Biomed. Anal.* 41 676–681. 10.1016/j.jpba.2006.01.011 16473490

[B37] Raza K. Thotakura N. Kumar P. Joshi M. Bhushan S. Bhatia A. (2015). C60-fullerenes for delivery of docetaxel to breast cancer cells: a promising approach for enhanced efficacy and better pharmacokinetic profile. *Int. J. Pharm.* 495 551–559. 10.1016/j.ijpharm.2015.09.016 26383841

[B38] Ren G. Liu D. Guo W. Wang M. Wu C. Guo M. (2016). Docetaxel prodrug liposomes for tumor therapy: characterization, in vitro and in vivo evaluation. *Drug Deliv.* 23 1272–1281. 10.3109/10717544.2016.1165312 26965023

[B39] Sansone F. Baldini L. Casnati A. Ungaro R. (2010). Calixarenes: from biomimetic receptors to multivalent ligands for biomolecular recognition. *New J. Chem.* 34 2715–2728. 10.1039/c0nj00285b

[B40] Shaw A. T. Kim D. W. Nakagawa K. Seto T. Crino L. Ahn M. J. (2013). Crizotinib versus chemotherapy in advanced ALK-positive lung cancer. *N. Engl. J. Med.* 368 2385–2394. 10.1056/NEJMoa1214886 23724913

[B41] Skiba M. Wouessidjewe F. Puisieux F. Duchene D. Gulik A. (1996). Characterization of amphiphilic ß-cyclodextrin nanospheres. *Int. J. Pharm.* 142 121–124. 10.1016/0378-5173(96)04653-4

[B42] Stella V. J. He Q. (2008). Cyclodextrins. *Toxicol. Pathol.* 36 30–42. 10.1177/0192623307310945 18337219

[B43] Swain S. M. Baselga J. Kim S. B. Ro J. Semiglazov V. Campone M. (2015). Pertuzumab, trastuzumab, and docetaxel in HER2-positive metastatic breast cancer. *N. Engl. J. Med.* 372 724–734. 10.1056/NEJMoa1413513 25693012 PMC5584549

[B44] Sweeney C. J. Chen Y. H. Carducci M. Liu G. Jarrard D. F. Eisenberger M. (2015). Chemohormonal therapy in metastatic hormone-sensitive prostate cancer. *N. Engl. J. Med.* 373 737–746. 10.1056/NEJMoa1503747 26244877 PMC4562797

[B45] ten Tije A. J. Verweij J. Loos W. J. Sparreboom A. (2003). Pharmacological effects of formulation vehicles : implications for cancer chemotherapy. *Clin. Pharmacokinet.* 42 665–685. 10.2165/00003088-200342070-00005 12844327

[B46] Wang D. Wang T. Xu Z. Yu H. Feng B. Zhang J. (2016). Cooperative treatment of metastatic breast cancer using host-guest nanoplatform coloaded with docetaxel and siRNA. *Small* 12 488–498. 10.1002/smll.201502913 26662850

[B47] Wang L. Li L. L. Fan Y. S. Wang H. (2013). Host-guest supramolecular nanosystems for cancer diagnostics and therapeutics. *Adv. Mater.* 25 3888–3898. 10.1002/adma.201301202 24048975

[B48] Weeden C. Hartlieb K. J. Lim L. Y. (2012). Preparation and physicochemical characterization of a novel paclitaxel-loaded amphiphilic aminocalixarene nanoparticle platform for anticancer chemotherapy. *J. Pharm. Pharmacol.* 64 1403–1411. 10.1111/j.2042-7158.2012.01518.x 22943171

[B49] Wilks T. R. Bath J. de Vries J. W. Raymond J. E. Herrmann A. Turberfield A. J. (2013). “Giant surfactants” created by the fast and efficient functionalization of a DNA tetrahedron with a temperature-responsive polymer. *ACS Nano* 7 8561–8572. 10.1021/nn402642a 24041260

[B50] Yin J. J. Zhou Z. W. Zhou S. F. (2013). Cyclodextrin-based targeting strategies for tumor treatment. *Drug Deliv. Transl. Res.* 3 364–374. 10.1007/s13346-013-0140-4 25788282

[B51] Youm I. Yang X. Y. Murowchick J. B. Youan B. B. (2011). Encapsulation of docetaxel in oily core polyester nanocapsules intended for breast cancer therapy. *Nanoscale Res. Lett.* 6:630 10.1186/1556-276X-6-630 PMC3292599 22168815

[B52] Yu X. Yue K. Hsieh I. F. Li Y. Dong X. H. Liu C. (2013). Giant surfactants provide a versatile platform for sub-10-nm nanostructure engineering. *Proc. Natl. Acad. Sci. U.S.A.* 110 10078–10083. 10.1073/pnas.1302606110 23716680 PMC3690883

[B53] Zhang L. Zhang N. (2013). How nanotechnology can enhance docetaxel therapy. *Int. J. Nanomed.* 8 2927–2941. 10.2147/IJN.S46921 PMC3742154 23950643

[B54] Zhao P. Astruc D. (2012). Docetaxel nanotechnology in anticancer therapy. *Chem. Med. Chem.* 7 952–972. 10.1002/cmdc.201200052 22517723

[B55] Zhou C. K. Check D. P. Lortet-Tieulent J. Laversanne M. Jemal A. Ferlay J. (2016). Prostate cancer incidence in 43 populations worldwide: an analysis of time trends overall and by age group. *Int. J. Cancer* 138 1388–1400. 10.1002/ijc.29894 26488767 PMC4712103

